# Retraction: MiRNA-200b regulates RMP7-induced increases in blood-tumor barrier permeability by targeting RhoA and ROCKII

**DOI:** 10.3389/fnmol.2025.1673270

**Published:** 2025-07-31

**Authors:** Frontiers Editorial Office

The journal retracts the February 5th 2016 article cited above.

Following publication, concerns were raised regarding the image integrity in the article. It was found that the images in Figure 7 were duplicated. An investigation was conducted in accordance with Frontiers' policies. The authors failed to provide a satisfactory explanation and as a result, the conclusions of the article have been deemed unreliable and the article has been retracted.

This retraction was approved by the Chief Editors of Frontiers in Molecular Neuroscience and the Chief Executive Editor of Frontiers. The authors do not agree to this retraction.

Frontiers would like to thank Elisabeth Bik for contacting the journal regarding the published article.

